# Quinine in Chorea

**Published:** 1895-04

**Authors:** 


Quinine in Chorea.—Knapp (*Boston Medical and Surgical Reporter*, February 28, 1895) reports eight cases of chorea in which quinine was administered in doses of from six to eighteen grains a day, the doses being somewhat smaller than those recommended by Dr. Wood. In addition to the quinine the ordinary hygienic measures and regimen ordinarily recommended in chorea were employed. No other medicinal treatment was given, not even cod-liver oil or any malt preparation. The author sums up the results as follows:

"In one case there was complete recovery in a week after treatment was begun, and in a second in three weeks, but although this is a rather short time, it is not very remarkable for chorea to recover in that period. The third case recovered after being under treatment ten weeks. In five cases the quinine treatment proved ineffectual, and arsenic was substituted, with distinct benefit in the majority of cases. With the exception of one case, which recovered in a week, the results are neither remarkable nor satisfactory."

The writer does not believe that the use of quinine in chorea can be justified on the hypothesis of its action in stimulating the inhibitory motor functions of the spinal cord, and believes that the good it accomplishes results from tonic properties or to some influence which it exerts on the toxine of the disease.

Chorea, he asserts, cannot be regarded as a spinal disease. The pronounced cerebral symptoms, and the distribution of choreic movements, which are so often found to affect one side or even one limb predominantly, would of themselves point to the cerebral origin of the disease, even without the evidences afforded by autopsies, which have shown the most marked changes in the cerebral cortex, although the basal ganglia and the cord itself have not been exempt. Choreic movements are not uncommon as a result of structural brain-disease, but they are rarely, if ever, seen as a symptom of disease of the cord. In canine chorea, on the other hand, which is not proven to be the same as human chorea, the changes are chiefly in the cord.

The hypothesis, the writer states, on which quinine is recommended for the treatment of chorea seems to him untenable, and the therapeutic results are based thus far on too few cases to warrant us in ascribing greater virtues to this drug than to arsenic.

